# Shiftless, a Critical Piece of the Innate Immune Response to Viral Infection

**DOI:** 10.3390/v14061338

**Published:** 2022-06-20

**Authors:** William Rodriguez, Mandy Muller

**Affiliations:** Department of Microbiology, University of Massachusetts Amherst, Amherst, MA 01003, USA; williamrodri@umass.edu

**Keywords:** C19ORF66, FLJ11286, shiftless, SVA-1, RyDEN, IRAV, ISG, innate immune response, RNA stability, translation, RNA granules, ribosomal frameshift

## Abstract

Since its initial characterization in 2016, the interferon stimulated gene Shiftless (SHFL) has proven to be a critical piece of the innate immune response to viral infection. SHFL expression stringently restricts the replication of multiple DNA, RNA, and retroviruses with an extraordinary diversity of mechanisms that differ from one virus to the next. These inhibitory strategies include the negative regulation of viral RNA stability, translation, and even the manipulation of RNA granule formation during viral infection. Even more surprisingly, SHFL is the first human protein found to directly inhibit the activity of the -1 programmed ribosomal frameshift, a translation recoding strategy utilized across nearly all domains of life and several human viruses. Recent literature has shown that SHFL expression also significantly impacts viral pathogenesis in mouse models, highlighting its in vivo efficacy. To help reconcile the many mechanisms by which SHFL restricts viral replication, we provide here a comprehensive review of this complex ISG, its influence over viral RNA fate, and the implications of its functions on the virus-host arms race for control of the cell.

## 1. Introduction

To overcome viral infection and ensure survival, cells have evolved a robust and efficient array of antiviral defenses. These defenses manifest as complex signaling cascades tied to equally elaborate shifts in cellular gene expression to regain control of cell fate. While the sensing of viral products—nucleic acids and proteins—is an essential first step in this process, the true sword of the intrinsic immune response are the hundreds of interferon stimulated genes (ISG) that work to establish an anti-viral state [1]. Each of these ISG target distinct steps of viral infection from viral entry and viral gene expression to virion assembly and egress (reviewed here [1]). Given the stunning diversity among viruses, ISGs are correspondingly adaptable at targeting multiple steps simultaneously to efficiently stamp out infection. One such ISG, *C19orf66* (herein referred to as Shiftless (SHFL)), has proven to be among the most versatile and yet still enigmatic anti-viral host factors identified.

An increasing number of large-scale screens have independently identified SHFL as both an anti-viral factor and downstream effector of the Type I, II, and III interferon response to viral infection [2,3,4,5,6,7,8,9]. Some studies have gone even further to characterize SHFL as a potent, broad-spectrum restriction factor for a growing list of DNA, RNA, and Retroviruses (Summarized in Table 1) [8,10,11,12,13,14,15,16,17,18,19,20]. Many ISGs have evolved to target viral gene expression as it is often the primary battleground that nearly every viral replication steps depend upon. However, while a few multifunctional ISGs have been described, they rarely demonstrate the same breadth of restriction mechanisms as SHFL. So far, for each virus that SHFL has been shown to restrict, a novel mechanism has been described. Here, we provide a comprehensive review of the many mechanisms by which SHFL restricts viral infection, the consequences of its many functions on the regulation viral and host RNA fate, and the implications of SHFL role as a critical piece of the innate immune response to viral infection. 

## 2. Shiftless: An ISG of Many Names

Located on chromosome 19 (NC_000019.10), *C19orf66* is an eight exon long gene that encodes four isoforms, with the full-length transcript (C19ORF66-201) (1928 bp) encoding a 291 amino acid (aa) protein of 33 kD molecular weight, C19ORF66. In nearly every context, this protein was identified as a broad-spectrum anti-viral factor whose expression is upregulated in response to Type I interferon (IFN) induction and generally viral infection. From these studies, several unique identifiers have been attributed to the *C19orf66* gene product including FLJ11286 (genomic location), Repressor of Yield of Dengue Virus (RyDEN), Suppressor of Viral Activity-1 (SVA-1), Interferon Regulated Anti-Viral gene (IRAV), and more recently Shiftless (SFL/SHFL) with SHFL finally approved for its official gene symbol [10,11,12,13,14,15,16,17,18,19,20]. SHFL expression can often vary dramatically between cell types as demonstrated by Balinsky and colleagues [11]. Among these cell lines were several immune cell types including monocytes, U937 monocytes, and Daudi B-lymphoblast cells that demonstrated significant SHFL protein expression upon IFN-B treatment. These observations are in-line with previous observations of differences in both IFN induction and ISG expression among diverse cell lines [21,22,23]. In terms of tissue-level expression of SHFL, there is a significant level of RNA detected in liver tissues but a low to moderate reported protein expression across various other tissues [24]. The apparent upregulation of SHFL in the liver coincides with its reported induction in response to Hepatitis C virus (HCV) infection. Of note, there is also a correspondingly low level of SHFL protein in the liver which could suggest significant post-transcriptional regulatory mechanisms that restrict the expression of SHFL outside the context of viral infection, perhaps due to cytotoxicity as has been previously reported [13].

While SHFL is indeed an ISG, the factors that govern its transcription have only recently been explored. For example, Wang, Hua and colleagues found SHFL to be a critical piece of a larger anti-viral pathway regulated by Early growth response gene 1 (ERG1) DNA binding protein and cellular transcription factor [18]. Wang, Hua and colleagues found that ERG1 was found to suppress the replication of PEDV, a pathogen of suckling pigs with >80% mortality rate across the Americas [25]. Upon further investigation, ERG1 was shown to regulate the expression of SHFL (here referred to as IRAV) by directly binding to the SHFL core promoter region (positions −61 to −1). Several other transcription factors were mapped to the SHFL promoter including KLF4, KLF5, and SP1-3. However, further studies are required to ascertain the relevant SHFL transcription factors across the various viral infections contexts it has been found in.

**Figure 1:**
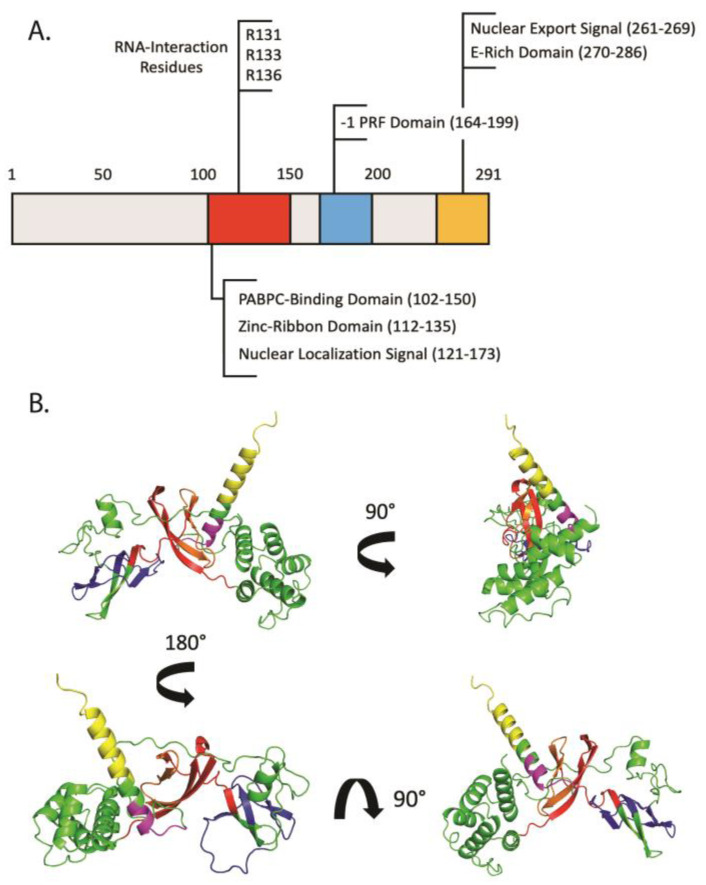
Shiftless Protein Structure. Shiftless is a 291aa long protein of 33 kDa molecular weight. (**A**) Highlighted in Red is the PABPC-binding domain (PABPC-BD) which also encompasses the Zinc-Ribbon Domain (112–135) and the Nuclear Localization Signal (121–173). Highlighted in Blue is the -1 Programmed Ribosomal Frameshift (PRF) (169–199). Highlighted in Yellow is the C-terminal domain containing the Glutamic Acid (E)-Rich Domain (270–286) and the Nuclear Export Signal (261–269). (**B**) Alphafold2 predicted protein structure of Shiftless. The PABPC-BD, -1PRF Domain, and the C-terminal Domain are Red, Blue, and Yellow respectively. Also highlighted in orange is the Nuclear Localization Signal and in pink is the Nuclear Export Signal [26,27].

The predicted protein structure of SHFL shares little sequence homology with other human proteins. From Jarred structural prediction by Suzuki and colleagues and our own Alphafold2 structural prediction, SHFL consists of eight α-helices, seven β-strands, a zinc-ribbon motif (aa 112–135), a coiled-coil motif (aa 261–285), a nuclear localization signal (NLS) (aa 121–173), a nuclear export signal (NES) (aa 261–269), and lastly a Glutamic acid (E) rich motif in the C-terminus [10] (Figure 1A). Apart from secondary structure predictions, SHFL also has two validated functional domains which we define here as the PABPC-binding domain (PABPC-BD) (aa 102–150) and -1PRF domain (aa 164–199), both of which will be discussed in more detail in later sections [10,13].

Several groups have hypothesized, although not experimentally validated, that SHFL has the capacity to dimerize with itself via its putative C-terminal coiled-coil motif. These higher order SHFL complexes would then be more readily able to bind its mRNA and ribosomal subunit targets to facilitate its regulatory functions relative to translation. Interestingly, none of the top five ranked SHFL Alphafold2 predicted structures reflect a coiled-coil motif, rather, a single alpha helix was predicted to form across the far C-terminal end of SHFL (Figure 1B). This structural prediction would in fact still support the idea of a SHFL dimerization, as the two C-terminal coils could in fact interact with one another to bridge the interaction in place of a coiled-coil motif. Importantly, this domain of SHFL also contains the critical NES, and therefore several layers of SHFL function may be simultaneously tied to this region of the protein. Our current understanding of SHFL structure relative to its multitude of functions is undoubtedly complex. The overlap between NLS and NES signals with known functional domains, the capacity to dimerize, and the myriad of different functions all culminate into an exciting challenge for understanding SHFL as a protein moving forward. However, incredibly important milestones, such as distinct residues involved in RNA binding, a growing list of validated interactors and reports of multiple SHFL mechanisms acting simultaneously, continue to set a critical foundation for future SHFL structural studies.

In terms of biological function, SHFL itself remains rather enigmatic. To date, no enzymatic functions have been attributed to SHFL itself. As such, SHFL functionality has often been tied to its interactions with a diverse set of cellular proteins (Table 1). The SHFL interactome across various viral infection backgrounds is most often enriched in RNA-binding proteins, most notably Poly-A Binding Protein Cytoplasmic 1 (PABPC) and the Moloney Leukemia Virus 10 (MOV10) proteins, which will be discussed in more detail in Section 3. In-line with these observations, SHFL itself has been experimentally validated as a bonafide RNA-binding protein with a strong, yet non-specific affinity for ssRNA [11,13,17]. While specific RNA binding domains have yet to be fully mapped out, recent in vitro work by Napthine and colleagues demonstrated that three arginine residues (R131, R133, and R136, Figure 1) within the predicted zinc-finger motif are critical for SHFL RNA binding [17]. This observation is further reinforced by the findings of three other SHFL studies that also mutagenize the SHFL NLS at the same three arginine residues albeit with additional mutated residues in select cases [10,14,19]. Furthermore, it appears that the binding interaction between SHFL and RNA-binding proteins (RBP) such as PABPC substantially enhances the binding affinity between SHFL and its target RNA [10]. These are rather surprising observations in consideration of findings by Wang and colleagues which identified an entirely separate domain of SHFL, here referred to as the -1PRF domain, that appeared to be necessary for its interaction with target RNA [13]. Thus, while the zing-finger motif/NLS of SHFL is demonstrably critical for SHFL RNA binding, it will be important moving forward to investigate potential synergistic functions across these seemingly disparate SHFL domains that could explain these complex observations of SHFL functionality.

## 3. Shiftless and Viral RNA Fate

The life of an RNA from the moment of transcription, execution of its functional output (i.e., translation), and unto its eventual decay, is collectively referred to as RNA fate [28,29,30]. While transcription and translation are considered the cornerstones of gene expression, RNA fate is undoubtedly the linchpin that inexorably ties these two processes together. Studies across years of RNA biology have uncovered hundreds of positive and negative feedback loops that allow the cell to finely tune gene expression in response to environmental challenges, especially that of viral infection [31,32,33,34,35]. This interconnectedness presents a significant challenge when investigating the mechanisms of RNA-binding proteins such as SHFL, particularly in the context of virus replication, which almost always coincides with dramatic alterations to the global RNP landscape. In this section, we will review the relationship between SHFL and its capacity to influence RNA fate during viral infection.

### 3.1. SHFL and Viral RNA Translation

In 2016, Suzuki and colleagues set out to identify novel anti-viral factors that are upregulated in response to type I IFN treatment and are refractory to Dengue Virus (DENV) infection [10]. DENV belongs to Flaviviridae, a large family of single-stranded positive-sense RNA viruses. Like most flaviviruses, following receptor meditated endocytosis, DENV replication takes place exclusively in the cytoplasm. DENV proteins work in concert to execute viral replication while simultaneously rewiring host cell organization and restricting the activation of the innate immune response [36]. Striking back against this manipulation, a plethora of ISG including IFIT2, IFIT1, ISG15 and TRIM69 have been shown to restrict flavivirus replication [37,38,39]. From their screen, Suzuki and colleagues identified yet another ISG, *C19orf66* (referred to as RyDEN in the study), as capable of conferring greater survivability to DENV infected cells and is upregulated in response type I, II, and III interferon treatment. Upon further investigation, they next demonstrated using a knockdown approach that steady-state levels of SHFL are sufficient in restricting the replication of all four DENV serotypes. Further investigation into the SHFL inhibitory mode of action revealed that SHFL expression significantly restricted the accumulation of DENV RNA in infected cells between 18 and 24 h post infection. A significant reduction in the amount of negative sense RNA also occurred in the same time frame. Compounding this, Suzuki and colleagues would be the first to demonstrate that loss of SHFL expression via knockdown (KD) results in catastrophic loss of IFN-mediated protection of the host cell upon DENV infection. This evidence highlights that SHFL is a vital piece of the innate immune response to DENV infection in human cells. To facilitate this restriction of DENV, SHFL was shown to interact with La-related protein 1 (LARP1) and PABPC, two RBP that are both known to regulate mRNA stability and the cellular translation apparatus (both extensively reviewed here [40]). Interestingly, in this same study, Suzuki and colleagues showed that both PABPC and LARP1 act as pro-viral factors during DENV infection as previously reported [41]. This could suggest that SHFL repurposes both proteins toward antiviral functions during flavivirus infection. Exploring the SHFL structure further, the authors next mapped SHFL anti-viral capacity to its NLS located within the PABPC-BD. Loss of this domain leads to an inability of SHFL to impact DENV replication and binding to PABPC as per its namesake. Recent work by Napthine and colleagues has shown that mutation of three arginine residues to alanines (R131A, R133A, and R136A, Figure 1) within the NLS results in the loss of SHFL RNA binding capacity [17]. One key difference to this study, however, is that Suzuki and colleagues also mutagenized several other arginine residues throughout the PABPC binding domain. Since Suzuki and colleagues showed that SHFL itself binds to the 3′UTR of DENV genomic RNA (gRNA), this NLS mutant thus proved that SHFL can bind viral RNA independently of PABPC, albeit with less affinity. Given SHFL ability to bind DENV gRNA, the authors next explored SHFL’s possible influence over DENV translation. Interestingly, SHFL stringently restricted the translation of DENV but did not significantly impact global cellular translation of the host. Thus, in their final model, Suzuki and colleagues concluded that through its binding to DENV 3′UTR, SHFL can restrict its translation while also possibly triggering viral RNA degradation.

Excitingly, Hanners and colleagues recently studied the in vivo efficacy of SHFL and broadly addressed its capacity to restrict a wide range of positive and negative sense RNA viruses [16]. In previous screens Hanners and colleagues utilized large lentiviral libraries of ISG to identify ISG with strong anti-viral activity against the flavivirus West Nile Virus (WNV), measuring WNV infectivity for each ISG tested. However, several ISG (including SHFL) were incompatible with lentivirus production and thus they applied a transient expression system to assess the antiviral capacity of each ISG examined in a one-gene to one-well tissue culture format. Among roughly 52 candidate genes, they found that SHFL exhibited a potent anti-viral effect against WNV. When testing the specificity of the SHFL phenotype, they found that SHFL was able to restrict each member of the Flaviviridae family tested including Hepatitis C virus (HCV), Yellow Fever Virus (YFV), WNV, DENV, and Zika Virus (ZIKV) and several other positive sense RNA viruses including Equine arteritis virus (EAV), Coxsackievirus B3 virus (CVB3), Venezuelan Equine Encephalitis virus (VEEV), Sindbis virus (SINV), and O’nyong’nyong virus (ONNV). Surprisingly, they did not observe an effect of SHFL on the infectivity of the human coronaviruses OC43 and SARS-CoV-2 or five negative sense RNA viruses including Vesicular stomatitis virus (VSV), parainfluenza virus 3 (PIV3), respiratory syncytial virus (RSV), measles (MV), or IAV. Given the breadth of SHFL among positive sense RNA viruses, this lack of an impact on negative sense RNA viruses most likely represents a staunch evolutionary divergence in the SHFL mechanism relative to differences in viral replication strategies. As has been posited previously by Suzuki and colleagues, this could boil down to a difference between the RNP structures assembled on the viral genome and/or viral mRNA at the earliest stages of primary infection. Perhaps positive sense RNA viruses, readily translated, are more easily recognized by SHFL or rather SHFL is recruited to viral translation complexes that are recognized by specific SHFL co-factors and therein triggers viral RNA decay/translational arrest.

Hanners and colleagues further explored the mechanism by which SHFL restricts HCV and YFV replication and found that, similar to DENV, SHFL binds to viral RNA, does not affect primary translation, but was still capable of restricting later stages of viral infection. They also observed a distinct loss of HCV replication organelles which could explain the dramatic impact of SHFL on overall viral gene expression. Correspondingly, there was also a distinct restriction of dsRNA intermediates produced as byproducts on viral genome amplification. Lastly, Hanners and colleagues studied the impact of the loss of SHFL on infection by the flavivirus ZIKV and alphavirus SINV in mice lacking SHFL expression. SHFL knockout (KO) mice displayed a more rapid onset of symptoms and increased severity of clinical disease compared to control mice, highlighting the significant role SHFL plays in viral pathogenesis in an in vivo model. Interestingly, SHFL KO mice also exhibited enhanced inflammation and ZIKV titers in the brain and spinal cord but no observable dysregulation of cytokine levels. This suggests that the cause of lethality is likely due to increased viral replication in the central nervous system and not an effect on the overall immune response to ZIKV infection. Thus, SHFL may have a significant impact on the neuroinvasiveness of ZIKV and perhaps other neurotropic flaviviruses such as WNV and Japanese Encephalitis virus (JEV) [42,43]. Given the myriad of mechanisms described thus far, several possibilities present themselves. First, SHFL targeting of viral RNA may be governed by the expression/functionality of a diverse set of RBP that bind to and facilitate subsequent rounds of viral translation. Second, SHFL could either, as suggested by Balinsky and colleagues, re-localize viral RNA to sites of RNA decay (namely P-bodies) or recruit RBPs involved in RNA decay directly to the viral genome. Lastly, SHFL could be acting on pathways completely independent of viral products as has been suggested recently by Kinast and colleagues regarding the biophysical formation of HCV replication compartments, known as the HCV membranous web, following infection. This observation was also observed by Hanners and colleagues with YFV by electron microscopy [14]. Much work remains to parse out each of these different possibilities, a challenge that mirrors the interconnectedness of RNA stability and translation during viral infection.

### 3.2. Shiftless and Viral RNA Stabillity

While each of the studies discussed thus far identified SHFL as a downstream effector of the IFN response, not all studies of SHFL study discovered it within these confines. In 2019, our group set out to investigate the back-and-forth struggle between herpesviruses and their hosts for control of cellular mRNA stability during viral infection [13]. During lytic replication, herpesviruses rapidly seize control of the cellular gene expression apparatus by triggering a massive cellular RNA decay event termed “host-shutoff” [44,45,46]. This global RNA decay is orchestrated within the cytoplasm by a single viral endoribonuclease. This strategy is surprisingly well conserved among related alpha- and gammaherpesviruses, including Kaposi’s sarcoma-associated herpesvirus (KSHV) ORF37 (SOX), Herpes Simplex virus-1 (HSV-1) vhs, Epstein–Barr Virus (EBV) BGLF5, and the Murine Herpesvirus 68 (MHV68) muSOX [47,48,49]. There are also several RNA viruses that have evolved the same host-shutoff strategy, including several human coronaviruses and influenza A virus (IAV) [50,51,52]. Each of these viral endonucleases, though diverse in mechanism, ultimately trigger the internal cleavage of messenger RNA (mRNA) targets, rendering these transcripts susceptible to both 3′ to 5′ as well as 5′ to 3′ directional RNA decay machinery such as degradation by the major cellular exonuclease XRN1 [45]. During KSHV infection, the viral endonuclease SOX is responsible for the degradation of about 70% of the host transcriptome, profoundly altering the host gene expression landscape [12]. Despite the global efficiency of host-shutoff, there remains a pool of cellular mRNAs that we and others have shown evade cleavage by SOX [50,53,54,55]. Among these “escapees” are those that actively evade SOX cleavage via an RNA element located within their 3′ UTRs that we refer to as the “SOX Resistant Element” or SRE [54]. In our study, to identify these “escapee” mRNA transcripts containing SRE or SRE-like elements, we used a comparative RNA-seq approach on cells individually expressing several herpesviral RNA endonucleases (KSHV SOX, MHV68 muSOX, EBV BGLF5, HSV-1 vhs). As expected, there was a significant number of gene downregulated by the expression of each endonuclease ranging from 55 to 70% of total mRNA degraded. Next, using hierarchal clustering on the expression data, we identified a cluster of 75 transcripts that evaded cleavage by all four of the endonucleases tested. Among these transcripts, we identified the SHFL mRNA (referred to as C19ORF66 in this study) and confirmed that it can efficiently evade cleavage. We next showed that SHFL expression climbs over the course of KSHV lytic replication and is predominately cytoplasmic as shown by cell fractionation and in line with previous observations by Suzuki and colleagues [10]. Given that SHFL previously demonstrated anti-viral capacity for DENV, we next found that SHFL also restricts KSHV infection, impacting nearly every step of the viral life cycle following lytic reactivation. The presence of an SRE on the SHFL mRNA coupled with its essential place in the innate immune response point starkly to a strong evolutionary imperative for its expression during viral infection. However, whether this impact on viral RNA stability is direct or indirect remains an important open question for our group.

### 3.3. Shiftless and RNA Granules during RNA Virus Infection

One pattern that emerges among studies of the SHFL interactome is that it is consistently dominated by constituents of phase-separated RNA granules [10,11,14,20]. RNA-granules are membrane-free, phase-separated ribonucleoprotein (RNP) complexes that function in the storage, translational arrest, and/or degradation of RNA throughout the cell [55,56,57,58]. These granules vary in size, shape, and overall function between the nucleus and the cytoplasm [59,60]. In the cytoplasm, two distinct granule types have gained increasing attention in the past 15 years; these include Processing bodies (P-bodies) and Stress Granules (SG). In a broader biological context, SGs and P-bodies are conserved across all eukaryotes and have been implicated in multiple processes ranging from stem cell differentiation to cancer development [61,62,63,64]. An important distinction between these two granule types is that SG are exclusively stress induced, while P-bodies are constitutive in some cell lines but increase in size and number in response to stress. Generally, it is accepted that SG are sites of translational arrest while P-bodies are sites of RNA-storage and decay. However, these functions relative to the phenotypes that regulate their formation remain an active area of research and is often contested [65,66,67,68,69]. Interestingly, the formation of both SG and P-bodies are also induced during viral infection by a diverse array of viruses [70,71,72,73,74]. A growing number of studies have begun to find a range of anti-viral roles for RNA granules during viral infection, many of which often link back to the translational arrest or degradation of viral RNA at these sites. However, the extent to which these cytoplasmic RNP granules facilitate anti-viral function or in some cases are coopted by viruses to facilitate replication remains to be explored [75,76,77,78]. It is important to note that while the function of RNA granules has been actively contested in the literature, it is generally agreed that their formation is still reflective of broader changes in gene expression. P-bodies for example are constitutively formed in most human cell types and are often a consequence of the activity of RNA-induced silencing complexes (RISC) which mediate the translational silencing of cellular transcripts [79,80,81]. SG on the other hand are often formed as a direct consequence of cellular translational arrest, a phenotype that can be both naturally and artificially induced with chemical/environmental stimuli such as Sodium Arsenate and cold shock [82,83,84]. In either case, RNP granules allow the cell a substantial level of control over gene expression, acting as “membraneless organelles” that allow for the fine-tuning of RNA stability and protein translation in response to challenges to cell health.

The next SHFL study by Balinsky and colleagues (here referred to as IRAV) would go on to verify that SHFL expression is upregulated in response to DENV infection [11]. They first demonstrated that SHFL itself is an ISG whose expression is coupled to the canonical IFN-induced ISGF3 pathway in an IFN-β dependent manner. It was then shown that SHFL restricts the replication of both DENV and Encephalomyocarditis virus (EMCV), drastically decreasing viral titers and viral RNA levels. To dig further into the mechanism of DENV/EMCV restriction, Balinsky and colleagues next showed that SHFL, whose steady-state localization is often diffusely cytoplasmic, is re-localized to the DENV replication complex, co-localizing with the viral proteins NS3 and NS4A, both of which are essential to the capping and amplification of DENV gRNA. Mirroring findings by Suzuki and colleagues, SHFL was also shown to interact with several known RBP that play critical roles in RNA stability including the RNA helicases MOV10, UPF1, and AU-rich element (ARE) mRNA binding protein HuR [85,86,87]. Given that SHFL interactors are predominately RNP-granule constituents, Balinsky and colleagues proceeded to investigate whether SHFL localizes to P-bodies in response to IFN treatment. Interestingly, they found that SHFL does in fact re-localize to P-bodies in IFN-treated cells. This a particularly interesting observation considering this experiment was performed in the absence of DENV infection. SHFL granularization in response to IFN may suggest SHFL influence over gene expression extends beyond viral infection. Exploring the SHFL interactome further, Balinsky and colleagues lastly confirmed the co-localization of SHFL with MOV10 in DENV infected cells, both of which re-localize to the DENV replication complex to restrict DENV replication. Importantly, several studies have previously shown that MOV10, a known P-body constituent, is itself an ISG with several anti-viral functions (reviewed in [88]). This represents one of the first studies to demonstrate a direct relationship between the anti-viral function of SHFL and phase-separated RNA granules, providing a novel link between the innate immune response to viral infection and RNP granule dynamics. 

Further reinforcing the ties between SHFL and RNA granules, recent work by Kinast and colleagues also demonstrated that SHFL can re-localize to SG upon HCV infection [14]. Interestingly, Kinast and colleagues also found that a Zinc-finger mutant was unable to restrict HCV infection. Given this RBP-enriched interactome, one outstanding question remains: what is the direct functional link between SHFL and RNA granules? More specifically, what benefit would RNA phase-separation have toward the capacity of SHFL to bind viral RNA or regulate viral gene translation? These are questions that unfortunately remain difficult to answer with the current lack of studies tracking the localization of SHFL relative to target RNA transcripts. In a similar vein, the size and shape of the granules reported to date also suggest that there may be human transcripts also caught by SHFL in its attempt to establish an anti-viral state. What these transcripts are, and whether they serve pro-viral roles, remains entirely underexplored.

### 3.4. Shiftless and RNA Granules during DNA Virus Infection

Recently, our group has investigated the mechanism by which SHFL restricts lytic viral gene expression [20] and explored the interactome of SHFL throughout KSHV infection. In line with previous reports, we have found a number of RNA binding proteins interacting with SHFL and we are now in process of further characterizing the relationship between SHFL and RNA granule formation during herpesviral infection. Several studies described throughout this review highlight SHFL’s capacity to influence and interface with various components of the translational machinery and as such its ability to induce translational arrest of viral/host transcripts could be reflected by its capacity to interface with SG components. Further investigation in this area will likely reveal novel facets of SHFL complex interplay with viruses.

To date, the interplay between these two arms of the anti-viral response, RNA granules and ISG, remains an active area of research. A growing number of studies have demonstrated that IFN signaling proteins often re-localize to SG upon viral infection [89,90]. Furthermore, several anti-viral proteins, some of which also ISG, are known constituents of RNP granules including FUS, MOV10, and more recently TDRD3 [90,91]. These observations all raise critical questions regarding SHFL: Does SHFL restrict the expression of select mRNA by re-localizing them to P-bodies as a part of the IFN response? If so, how does this related to SHFL disassembly of P-bodies when exogenously expressed? Do these target mRNA otherwise serve pro-viral roles? If so, what are these transcripts and what dictates their targeting by SHFL from cell type to cell type? Lastly, could these differences in targeting explain the expansive breadth SHFL modes of function? Complicating this further, work by Suzuki and colleagues also reports that SHFL had no impact on global gene translation, which is contradictory to what would be assumed of its impact on translation and SG localization. This could suggest that SHFL-containing granules, though reminiscent of SG in composition, may not reflect a global impact on translation but rather a localized accumulation of specific mRNAs. Nonetheless, these questions will be critical to assess moving forward, as RNA granules (and phase-separation itself) could be a critical missing link that may bridge several SHFL mechanisms.

## 4. Shiftless and the -1 Programmed Ribosomal Frameshift

While we still cannot fully mechanistically define the SHFL anti-viral strategies discussed thus far, most are similar to strategies employed by other ISG expressed in response to viral infection. However, in this section we will discuss a mechanism that is unique to SHFL and is especially effective against a subset of human viruses including retroviruses such as Human Immunodeficiency virus-1 (HIV-1), select flaviviruses such as WNV and JEV and, of recent notoriety, coronaviruses such as SARS-CoV-2 [92,93,94,95]. 

In 2019, a study by Wang, Xinlu and colleagues leveraged the anti-viral response to HIV-1 to identify novel host factors the modulate a viral translation strategy known as the -1 programmed ribosomal frameshift (-1PRF) [15]. The -1PRF is a translation-recoding strategy to expand genetic coding capacity, where an actively translating ribosomes slips back one nucleotide on select viral open reading frames, resulting in translation of a new reading frame (Reviewed Here [96]). The -1PRF signal itself is a *cis*-acting RNA element consisting of two RNA motifs: one is a heptameric slippery sequence X XXY YYZ, wherein X is any nucleotide; Y is A or U; and Z is A, U, or C. Prior to the shift in the -1 direction, the original frame (0 frame), are ordered as codons XXY and YYZ, but in the -1 frame, the reading frame is back shifted one nucleotide resulting in the codons XXX and YYY. The second RNA motif is an RNA secondary structure that serves as stimulatory signal to the ribosome known as an RNA pseudoknot downstream of the slippery sequence. During lytic replication, HIV-1 uses a -1PRF cue to control the ratio between the Gag and Gag-Pol polyproteins [97]. Several groups have shown that mutagenesis of this region can severely inhibit this frameshift which results in a stringent restriction of HIV-1 productive infection [98,99]. Using a screen for IFN-linked effectors modulating the -1-frameshift efficiency of Gag-Pol, Wang, Xinlu and colleagues identified SHFL, which displayed the highest inhibitory activity against the -1PRF reporter construct assayed. Thus, *C19orf66* was renamed “Shift-less” or Shiftless (SHFL). Upon further investigation, SHFL was shown to restrict HIV-1 replication by directly disrupting the balance between Gag and Gag-Pol expression in favor of a short-form premature translation termination (PMT) product. Furthermore, when testing the inhibitory capacity of SHFL against other -1PRFs, Wang, Xinlu and colleagues found that SHFL appears to be a broad inhibitor of the -1PRF for multiple retroviruses including Rous sarcoma virus (RSV), Mouse mammary tumor virus (MMTV), Human T-lymphotropic virus (HTLV), HIV-2, and SIV. Surprisingly, two notable human mRNAs, CCR5 and PEG10, also contain -1PRF signals that are also restricted by SHFL suggesting that SHFL may be the only known trans-acting human protein capable of restricting -1PRF signals [100,101]. To better understand the mechanisms by which SHFL restricts the -1PRF, Wang, Xinlu and colleagues next investigated SHFL interactome and found that SHFL directly interfaces with active ribosomes and target -1PRF bearing mRNAs. This binding of SHFL to -1PRF transcripts triggers a PMT event that leads to the enrichment of shortform products from the shorter open reading frame. The authors then posited that this inhibition may be due to a restriction of a non-canonical ribosome rotation by SHFL through recruitment of factors that facilitate PMT. In line with this, SHFL was found to bind to the ribosomal binding protein Eukaryotic polypeptide chain release factor 3 (eRF3) in complex with eRF1, which are both known to help facilitate ribosome rotation [102]. Thus, it was concluded that SHFL binds to -1PRF bearing mRNAs in response to ribosome stalling at the -1PRF element and therein triggers a PMT event that leads to the formation of the PMT product. This study marks SHFL as a unique human gene capable of regulating the -1 frameshift, further emphasizing the extraordinary versatility of this ISG.

Recent studies have also followed up on this unique feature of SHFL, testing its ability to modulate -1PRFs outside of retroviruses. A recent study by Sun and colleagues found that SHFL could restrict the -1PRF signal within SARS-CoV-2 genome in vitro. While this would imply that SHFL has the capacity to restrict SARS-CoV-2 replication, the recent report by Hanners and colleagues showed that SHFL was unable to restrict SARS-CoV-2 infection [16]. This could suggest that inhibition of -1PRF is not necessarily sufficient to restrict SARS-CoV-2 or that other confounding factors could prevent SHFL function during coronavirus infection in vivo. Fascinatingly, another recent study has also showed that -1PRF mechanisms can work in tandem with other SHFL anti-viral strategies to restrict flavivirus infection. In a study conducted by Yu and colleagues, SHFL was identified as a potent anti-viral ISG capable of restricting JEV, yet another neurogenic flavivirus [19]. However, surprisingly, this group reported that SHFL not only inhibited the -1PRF-mediated expression of NS1′ but also triggered the lysosomal degradation of JEV NS3 in a manner parallel to findings by Wu and colleagues for ZIKV NS3 [15]. Of note, the capacity of SHFL to modulate these -1PRF elements was mapped by Wang, Xinlu and colleagues to a C-terminal domain of SHFL (aa 164–199, labeled in Figure 1 as “-1PRF Domain”). SHFL lacking the -1PRF domain mirrors a true isoform of SHFL (C19orf66-209) which is expressed in select cell types but to date has displayed no attributable anti-viral function [13]. Interestingly, work by Napthine and colleagues, who demonstrated that bacterially expressed, purified SHFL can inhibit the -1PRF, also showed that this domain of SHFL is dispensable for its ability to restrict the -1PRF in vitro [17]. They also showed that SHFL binds indiscriminately to single stranded RNA substrates regardless of the presence or absence of the -1PRF. These findings directly contradict those made previously by Wang, Xinlu and Yu and colleagues more recently and therefore warrant further investigation. Taken together, with SHFL influence over viral RNA and viral protein stability, SHFL has emerged as a critical regulator of viral gene expression. However, observations of SHFL’s influence over human -1PRF signals, such as in CCR5 and PEG10, also suggest that the SHFL phenotype may have a much broader impact on cellular gene expression than previously hypothesized.

## 5. Conclusions

**Figure 2:**
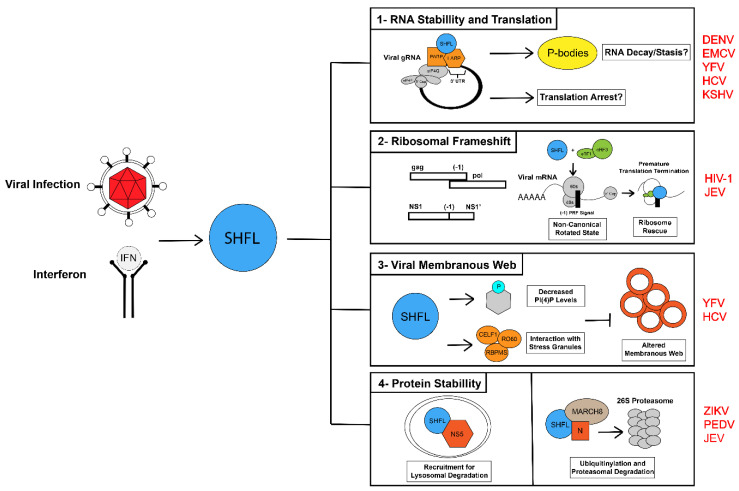
Shiftless Mechanisms of Restricting Viral Infection. Shiftless (SHFL) has been demonstrated to be a potent broad-spectrum anti-viral factor that is upregulated in response to viral infection and interferon signaling. SHFL restricts viral infection through several mechanisms summarized here: (1) SHFL directly interfaces with viral genomic RNA and viral mRNA and restricts viral gene expression at various stages between RNA stability and translation. For flaviviruses, SHFL binds to viral genomic RNA at the 3′ end and may relocalize it to Processing bodies to trigger RNA decay or directly interfere with polyprotein translation. (2) SHFL is one of the first human proteins shown to directly restrict the function of the -1 programmed ribosomal frameshift (-1PRF), a cis-RNA element that extends the coding capacity of several retro- and RNA viruses. The -1PRF signal triggers a non-canonical ribosome rotation, signaling the recruitment of SHFL and eukaryotic ribosome release factors (eRF1/eRF3), which synergistically halt and then trigger the premature release of the ribosome from frameshifting viral RNA. (3) For HCV and YFV, SHFL expression decreases the level of PI(4)P in the cell, a lipid precursor that directly contributes to the formation of the viral membranous web, a collection of reorganized lipid membranes that house viral replication compartments. SHFL was also shown to interact with and localize to Stress Granules during HCV infection. (4) Lastly, SHFL has been recently shown to bind to and trigger the degradation of select viral proteins through lysosomal or ubiquitination based pathways. Abbreviations: Shiftless (SHFL), Dengue virus (DENV), Encephalomyocarditis virus (EMCV), Yellow Fever virus (YFV), Hepatitis C virus (HCV), Kaposi’s sarcoma-associated herpesvirus (KSHV), Human Immunodeficiency virus 1 (HIV-1), Japanese Encephalitis virus (JEV), Zika virus (ZIKV), Porcine Epidemic Diarrhea virus (PEDV), Poly-A Binding Protein (PABP), La-associated RNA binding protein (LARP), eukaryotic Initiation Factor 4G (eIF4G), eukaryotic Initiation Factor 4E (eIF4E), Processing bodies (P-bodies).

Of the human ISG studied to date, few have demonstrated such extraordinary versatility in restricting viral infection as SHFL (Figure 2). While our present review has covered much of our current understanding of SHFL and viral infection, new studies continue to unveil even more complex mechanisms-of-action. Recent work by Wu and colleagues as well as Wang, Hua and colleagues has begun to tie SHFL function directly to the anti-viral capacity of autophagy and the ubiquitination pathways, respectively [15,18]. For certain viruses, as suggested by the work of Kinast and colleagues, these mechanisms may also be entirely distinct from those directly targeting viral replication products [14,16]. A more exciting possibility and a significant challenge to overcome moving forward will be to determine which of the many SHFL mechanisms are applicable from one virus to the next. Within these systems are their multiple strategies working simultaneously as seen with JEV [19]. While not often consistent from one infection context to the next, further investigation of how SHFL regulates both viral and host mRNA along the axis of RNA stability and translation will likely prove to be essential for one or more of the mechanisms discussed here. In line with this, understanding the relationship between SHFL and RNA granules may lead to novel insights into a broader influence of SHFL over global gene expression and how this relates to the establishment of an anti-viral state.

Given the breadth of its anti-viral function, SHFL also provides a wide-array of translational therapeutic opportunities. First, the SHFL mRNA is an SRE-bearing transcript and is therefore resistant to cleavage by multiple herpesviral endonucleases. Therefore, it is plausible that SHFL could be used directly or included in combination with other mRNA-based anti-viral therapies for viruses that utilize host-shutoff as a takeover strategy. The SRE element of SHFL can also be transposed to other non-escapee mRNAs and could confer its natural resistance to them, circumventing a significant layer of RNA stability concerns in RNA therapeutic formulation. Furthermore, given its capacity to potentially restrict the neuroinvasiveness of ZIKV, and its modest expression levels in neuronal tissues [24], continued studies of SHFL capacity to restrict other neurotropic viruses is assuredly warranted. It should also be noted that SHFL capacity to localize to and perhaps even influence the formation of RNA granules could also be a useful tool for modulating the expression of select transcripts, particularly those that are known to localize to specific cytoplasmic granule types and play roles in cancer development, However, this will require significant further research relating the connection between SHFL and RNA granules to cell viability in various contexts.

Taken together, SHFL has proven to be a critical piece of the innate immune response to viral infection that absolutely warrants further study. In doing so, we will undoubtedly uncover further weaknesses within viral replication and better understand the complex web that ties the innate immune response to the fate of RNA during viral infection.

## Figures and Tables

**Table 1:** Summary of Shiftless Studies to-date. Table listing each of study of Shiftless and its capacity to restrict viral infection through diverse mechanisms, human protein interactions, and targets. Studies marked with * also examined the impact of Shiftless on multiple viruses in larger panels.

Authors	Viruses Studied	Restriction Strategy	Target	RNA Granule Association	Reference
Suzuki (2016)	DENV (Serotypes 1–4) *	Viral Translation, Viral RNA Stabillity	Genomic RNA	-	[10]
Xiong (2016)	HIV-1	Viral Gene Expression	p24	-	[8]
Balinsky (2017)	DENV and EMCV	Viral RNA Stabillity	Genomic RNA	Localization to P-bodies	[11]
Rodriguez (2019)	KSHV	Viral Gene Expression	Early/Delayed Early Genes	-	[12]
Wang (2019)	HIV-1	(-1) Frameshift	Ratio of Gag-Pol	-	[13]
Kinast (2020)	HCV	Viral Replication Compartment	Membranous Web, PI(4)P	Localization to Stress Granules	[14]
Wu (2020)	ZIKV	Lysosomal Degradation	NS3	-	[15]
Hanners (2021)	YFV and HCV*	Viral Gene Expression	Genomic RNA	-	[16]
Wang (2021)	PEDV	Ubiquitinylation-based Degradation	Nucleocapsid (N) Protein	-	[18]
Yu (2021)	JEV	(-1) Frameshift, Lysosomal Degradation	NS1′-NS1 ratio, NS3	-	[19]
Rodriguez (2022)	KSHV	Viral Gene Expression	ORF57	Restricts P-Body Formation, Stress Granule-like Densities	[20]
